# Ammonia Oxidizers in a Pilot-Scale Multilayer Rapid Infiltration System for Domestic Wastewater Treatment

**DOI:** 10.1371/journal.pone.0114723

**Published:** 2014-12-05

**Authors:** Yingli Lian, Meiying Xu, Yuming Zhong, Yongqiang Yang, Fanrong Chen, Jun Guo

**Affiliations:** 1 School of Biological Science & Engineering, South China University of Technology, Guangzhou, 510006, China; 2 Guangdong Institute of Microbiology, Guangzhou, 510070, China; 3 Guangzhou Institute of Geochemistry, Chinese Academy of Sciences, Guangzhou, 510640, China; CAS, China

## Abstract

A pilot-scale multilayer rapid infiltration system (MRIS) for domestic wastewater treatment was established and efficient removal of ammonia and chemical oxygen demand (COD) was achieved in this study. The microbial community composition and abundance of ammonia oxidizers were investigated. Efficient biofilms of ammonia oxidizers in the stationary phase (packing material) was formed successfully in the MRIS without special inoculation. DGGE and phylogenetic analyses revealed that proteobacteria dominated in the MRIS. Relative abundance of ammonia-oxidizing archaea (AOA) and ammonia-oxidizing bacteria (AOB) showed contrary tendency. In the flowing phase (water effluent), AOA diversity was significantly correlated with the concentration of dissolve oxygen (DO), NO_3_-N and NH_3_-N. AOB abundance was significantly correlated with the concentration of DO and chemical oxygen demand (COD). NH_3_-N and COD were identified as the key factors to shape AOB community structure, while no variable significantly correlated with that of AOA. AOA might play an important role in the MRIS. This study could reveal key environmental factors affecting the community composition and abundance of ammonia oxidizers in the MRIS.

## Introduction

Nowadays human activities harmfully affect limited freshwater resources. Freshwater resources on Earth are diminishing rapidly, making water resource conservation and regeneration a serious challenge for human beings. Efficient water reuse techniques play an important role in wastewater treatment. Intermittent infiltration systems are among the most promising systems due to their simplicity, reliability, low energy consumption and low cost. Such systems combine the complex effect of physical filtration, chemical reaction and biological transformation, thus can achieve high purification efficiency for domestic wastewater treatment. The biological transformation mainly refers to microbial nitrification and denitrification, is gaining more and more attention recently due to its significant contribution to the nitrogen removal in these systems [1].

Nitrification is the microbial oxidation of ammonia to nitrate via nitrite as intermediate, which plays an important role in the global nitrogen cycle and in controlling effluent toxicity in wastewater treatment [1]. Ammonia-oxidizing microorganisms (AOM), including ammonia-oxidizing archaea (AOA) and ammonia-oxidizing bacteria (AOB), are thought to be the main ammonia oxidizers. Both AOA and AOB contain ammonia monooxygenase (AMO) which catalyzes the first rate-limiting step of ammonia to hydroxylamine [2]. Autotrophic AOB including *Nitrosomonas communis*, *Nitrosococcus mobilis*, and *N. halophilus* affiliate with β- and γ-proteobacteria, had been considered to be the most important contributor to ammonia oxidation for a long time [3]. However, recent investigation found that AOA would also be responsible for nitrification. At present, thermophilic strains of AOA had been cultivated, such as *Candidatus Nitrosocaldus yellowstonii* and *Candidatus Nitrososphaera gargensis*, implying a broad distribution of ammonia oxidation among crenarchaeota, which reinvigorates the debate on the thermophilic ancestry of AOA [4], [5]. In certain environmental conditions, AOA even contribute more to microbial nitrification than AOB. Numerical dominance of archaeal over bacterial ammonia oxidizers in soil ecosystems indicated that crenarchaeota migh51ht be the most abundant AOM [6]. Exclusive growth of archaeal ammonia oxidizers revealed that ammonia oxidation under active nitrification condition was mainly due to AOA nitrification [5], [7].

Present reports showed that in wastewater treatment system (WWTS) physiological and ecological difference occurred among differences AOM genera and lineages in response to different environmental factors such as substrate concentration, temperature, salinity, pH, biogeography, and so on [8]–[10]. It can be expected that the AOM community structure also changes in the pilot-scale rapid infiltration system (MRIS) established in this article in response to different treatments. Hence, investigating the community structure of ammonia oxidizers in the MRIS induced by these environmental factors will improve our understanding about their roles in the nitrogen cycling in terrestrial ecosystems, and supply feedback to reevaluate WWTS. Present methods/techniques for investigation of AOM community structure consist of culture methods and biochemical techniques, etc. However, all culture methods are potentially selective and thus bear the risk of incomplete coverage of the actually existing bacterial diversity [11]. Furthermore, most AOM is difficult to cultivate in laboratory, which prevents its natural community structure analysis. Uncultured techniques based on *in situ* detection of 16S rRNA and *amo*A genes such as denaturing gradient gel electrophoresis (DGGE) can supply useful information about the microbial diversity in comparison to laboratory culture methods [12]. Moreover, quantitative analysis of the nitrifying indicator *amo*A genes by real-time quantitative polymerase chain reaction (qPCR) allows fast, sensitive, and simple inspection of variation of AOM abundance in WWTS [3].

In this study, we established a modified intermittent infiltration system named MRIS and evaluated its contaminant removal efficiency with several criteria including chemical oxygen demand (COD), NH_3_-N, NO_3_-N, pH, dissolved oxygen (DO), and total nitrogen (TN). Moreover, we analyzed the microbial community structure using DGGE profiles based on 16S rRNA gene and *amo*A gene sequences. Finally, we quantitated the *amo*A genes of different samples collected from different spots of the MRIS by qPCR technique, with the purpose of elaborating the correlation between the criteria detected and the microbial community.

## Materials and Methods

### Construction of the pilot-scale MRIS

**Figure 1:**
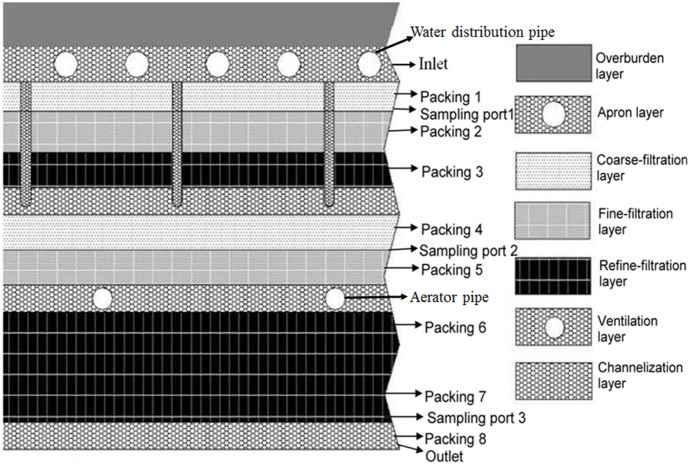
Structure of the filter sheet and sampling ports.

The MRIS was established outdoor and was operated nearby an apartment in the yard of Guangzhou Institute of Geochemistry (the field study did not involve endangered or protected species; location: latitude/longitude: 23.129163N/113.264435E; and no specific permissions were required for this location). Main part of the system is the filter sheet, which was stacked with sands of different diameters to form 11 layers in a leak proof cement pond. The total height of the filter sheet is 100 cm and length/width is 100 cm/100 cm. The crucial structure layers include the coarse-filtration layer (effective size d10 = 0.19, d60 = 0.61, uniformity coefficient = d60/d10 = 3.2), fine-filtration layer (effective size d10 = 0.17, d60 = 0.62, uniformity coefficient = d60/d10 = 3.6) and the refine-filtration layer (effective size d10 = 0.15, d60 = 0.62, uniformity coefficient = d60/d10 = 4.1). Thickness of packing 1 is 6 cm, packing 2 is 8 cm, packing 3 is 7 cm, packing 4 is 8 cm, packing 5 is 8 cm, total thickness of packing 6 and 7 is 25 cm, and packing 8 is 6 cm. Water distribution pipes were placed in the Apron layer and aerator pipes were placed in the Ventilation layer (Figure 1). The MRIS was fed with domestic wastewater but not fecal sewage, and was automatically operated. In order to prevent choking, wastewater was poured into a sedimentation tank via water distribution pipe before being pumped in to the filter system. Wastewater was intermittently pumped from the sedimentation tank into the filter, the pump was automatically turned on eight times per day and was operated for 27 min each time, with a total loading rate of 0.5 t/m^2^·d. The detail running times were 0∶00–0∶27, 3∶00–3∶27, 6∶00–6∶27, 9∶00–9∶27, 12∶00–12∶27, 15∶00–15∶27, 18∶00–18∶27, 21∶00–21∶27. The MRIS was aerated intermittently twice during the hydraulic retention time, 10 minutes each time through the ventilation layer (e.g. between 0∶27 to 3∶00, the MRIS was aerated in 1∶30–1∶40 and 2∶30–2∶40) (Figure 1).

### Sampling and chemical analysis

According to pre-experiments, the treatment efficiency of the MRIS reached steady state after being operated for 4 months. Four water sampling ports (Sampling port 1–3 and the Outlet) were set in different layers in the filter sheet (Figure 1). As microbial biomass varied considerably in the filter sheet, different volumes of water sample in different sampling ports were sampled to obtain approximately biomass. 300 mL raw wastewater, 500 mL, 1000 mL, 3000 mL, 5000 mL treated wastewater effluent from the Inlet (assigned as A1, similarly hereinafter), Sampling port 1 to 3 (sequentially A2–4), and the Outlet (A5) were collected in order (Figure 1). Triplicate water samples were collected eight times on the same day and were respectively mixed as one sample. Packing material was collected in different depths of the filter sheet, i.e., Packing 1 to 8 (assigned as P1–8 sequentially) (Figure 1). Water content of the packing material was measured within 24 h.

NH_3_-N was measured colorimetrically according to the Standard Methods [13]. NO_3_-N and NO_2_-N were determined with an iron chromatography (Shimadzu SCL-10ASP, Japan). The pH value was determined potentiometrically using a pH analyzer (Sartorius PB-20, Germany). The DO level was analyzed with a digital, portable DO meter (HQ30d, America).

### DNA extraction and PCR amplification

For DNA analysis, water samples were filtered respectively over 0.22 µm pore size polycarbonate membranes (45 cm×diameter), the filter cake were stored at −30°C until use. In order to get rid of big particles, all water samples were centrifuged at 500 rpm for 15 min at room temperature. The packing material was first washed by ultrapure water to remove humus, and then was shocked with glass bead to obtain microbial biomass. Genomic DNA was extracted from filter cake (about 0.2 g) or washed packing material (about 0.5 g) by UltraClean Soil DNA Kit (MoBio Laboratories, Solana Beach, CA, USA).

Nested PCR was conducted to increase the sensitivity of DGGE profile of 16S rRNA gene. In the first round, 27F/1492R primers were used according Brosius et al [14]. During the second round, 16S rDNA fragments were reamplified using bacterial primers 338F/534R [15]. For DGGE profile of *amo*A gene, bacterial *amo*A gene specific primers amoA-1F-26 GC (containing GC clamp) and amoA-2R [16], archaeal *amo*A gene specific primers arch-23F/arch-616R (both without GC clamp) were used [17]. The PCR mixture was prepared with 5 µL of 10×PCR buffer, 2 µL of dNTPs mixture (TaKaRa, China), 1 µL of each primer (10 µM), 1 µL of DNA extract, 0.5 µL of Taq polymerase, 1 µL of bovine serum albumin (BSA, 0.1%), and was adjusted to a final volume of 50 µL with ddH_2_O.

### DGGE profile of 16S rRNA and *amo*A genes

DGGE profile was carried out in 8% (w/v) polyacrylamide gels with a denaturing gradient of 35–75%, 0–45% and 40–70% for 16S rDNA V3 region PCR products, archaeal and bacterial *amo*A gene PCR products, respectively. Electrophoresis was performed in 1×TAE buffer at 60°C, 80 V for 12.5 h. Gels were stained for 20 min in 150 mL 1×TAE buffer containing 100 ng/mL Goldviewer dye. Visualization and digital photography was acquired with a CCD camera controlled by Quantity One software (Bio-Rad, USA) [16]. Major bands were excised and reamplified, PCR products were cloned into pMD19-T Simple Vector (TaKaRa, China). Four to six randomly selected clones containing correct insert size from each DGGE band were sequenced using M13–47 primer. Positive sequences were aligned using BLAST (http://blast.ncbi.nlm.nih.gov/Blast.cgi) and Clustal X 1.83. A phylogenetic tree was constructed using the Neighbor-Joining method with Jukes-Cantor correction by MEGA v4.0.2 software [18]. Robustness of tree topology was verified by calculating bootstrap values of 1000 replications for the Neighbor-Joining tree as previously described [19].

### qPCR analysis for AOB and AOA *amo*A gene

The abundance of AOB and AOA *amo*A genes was quantified with qPCR using previously described primers (amoA-1F and amoA-2R for AOB, amo196F and amo277R for AOA) [19], [20]. The reaction mixture contained 25 µL of 2×IQTM SYBR green supermix (TaKaRa, China), 10 pmol of each primer, and 20 µg BSA in a final volume of 50 µL. Amplification, detection, and data analysis were performed in triplicate using the Eppendorf MasterCycler ep Realplx^4^ system (Eppendorf, Germany). A control was always run with water as template instead of AOB or AOA DNA extract. Specific amplification of AOB or AOA *amo*A genes were confirmed by melting curve analysis always resulting in a single peak and by agarose gel electrophoresis. The fluorescence signal of the amplified DNA was used to quantify the concentration of AOB and AOA *amo*A genes [21]. Quantification was based on the comparison of the *C*_T_ value between samples and the calibration curve of *amo*A gene standard. The AOB and AOA numbers were calculated by assuming two or one *amo*A gene copy number(s) per cell, respectively [20], [22].

### Statistical analysis

SPSS 16.0 was used to evaluate the correspondence between the Shannon diversity index, environmental variables and *amo*A *gene* abundance. Bivariate followed by Pearson and two-tailed was used to check for the correlation coefficients, *p*<0.05 was considered to be statistically significant and *p*<0.01 was highly significant. To investigate the correlation between microbial community composition and environmental variables, CANOCO for windows (version 4.5) was used for multivariate statistical analysis. The relative intensity of each DGGE band represented microbial community composition of effluent. Detrended correspondence analyses (DCA) of DGGE band matrices indicated that the maximum of lengths of gradient was below 3, so redundancy analysis (RDA) was performed [23]. The significance of the relationship of environmental variables to the variation in microbial community composition was tested using Monte Carlo tests (999 unrestricted permutations, *p*<0.05).

### Nucleotide sequence accession numbers

The sequences reported in this study have been deposited in GenBank under the accession numbers of JQ963286 to JQ963324 for bacterial 16S rDNA V3 region, and KF460097 to KF460108 for *amo*A genes of AOB and AOA.

## Results

### Treatment performance of the MRIS

**Figure 2:**
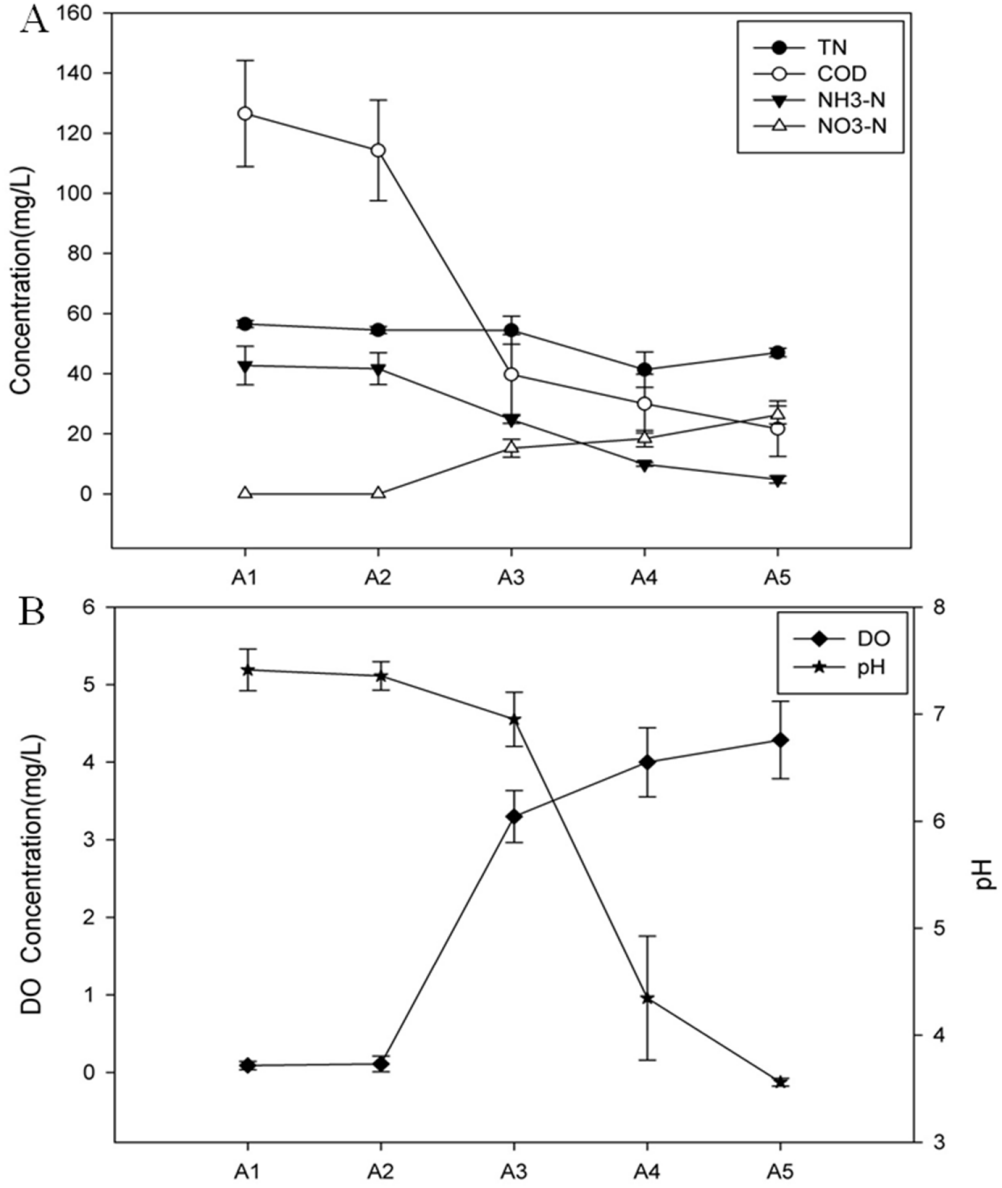
Performance of the MRIS for domestic wastewater treatment. (A) The concentration of chemical oxygen demand (COD), total nitrogen (TN), ammonia nitrogen (NH_3_-N), nitrate nitrogen (NO_3_-N); (B) Dissolved oxygen (DO) concentration (the right axis) and pH value (the left axis) in the Inlet, Sampling ports and the Outlet. A1–5: the Inlet, Sampling port 1–3, and the Outlet, correspondingly.

The constructed MRIS exhibited excellent performance without special inoculum. The loading burden could reach to 0.5 t/m^2^·d. As the system had been in steady state after running for 4 months, water quality could be regarded to be at the homogeneous level. To further assure the stability and representativeness of the analyses, water samples used were collected in triplicate and mixed as one final sample for all experiments, as indicated by the Sampling and chemical analysis above (see Materials and Methods). The structure of Ammonia-oxidizing microorganism community can be significantly affected by temperature in an infiltration bioreactor, and the DGGE fingerprints of AOM communities will consequently change according to the ambient temperature. However, in this study, the temperature of all samples could be treated as the same, as triplicate water samples were moderately and intermittently collected eight times on the same day and were respectively mixed as one sample. The pH value decreased from 7.41 to 3.56 along the depth of the filter sheet, which suggested that acidifying nitrification process occurred (Figure 2B). DO was only 0.9 mg/L in the upper layer of the filter sheet, while in the Outlet (A5) it increased dramatically to 4.29 mg/L (Figure 2B). The total removal ratio of COD and NH_3_-N in the MRIS were 82.8% and 88.7%, respectively (Figure. 2A). Between sampling port 1 (A2) and 2 (A3), COD removal ratios reached the highest (Figure 2A) and DO increased dramatically (Figure 2B). No or very low nitrite could be detected in raw wastewater (A1) and the ongoing-processed wastewater sampled from A2, while in the following sampling ports (A3 and A4) and the Outlet (A5), nitrate concentration increased evidently along the depth of the filter sheet (Figure 2A). All these results indicated that efficient microbial nitrification was achieved in this MRIS.

### Community diversity and distribution of AOB based on 16S rRNA gene

**Figure 3:**
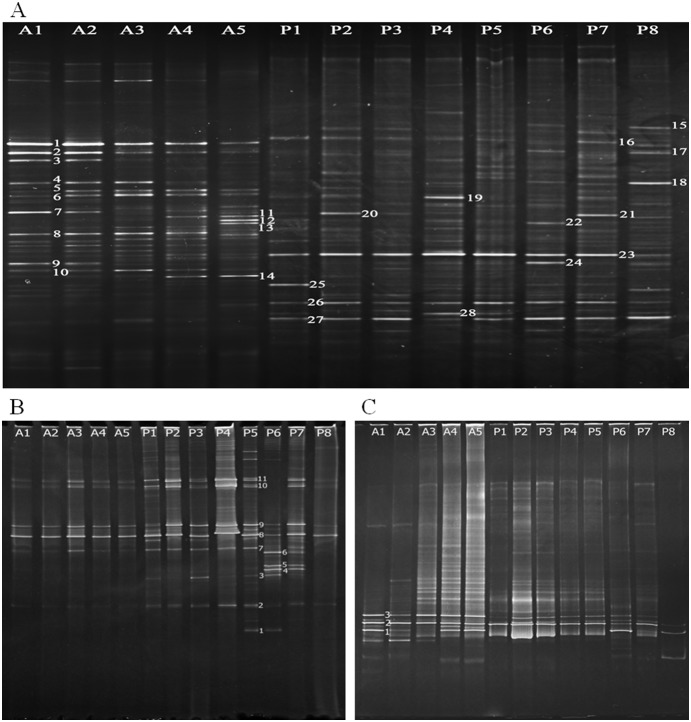
DGGE profiles of 16S rRNA and *amo*A genes. 16S rRNA genes (A), AOB *amo*A genes (B), and AOA *amo*A genes (C) were amplified from the flowing (A1–5) and the stationary phase (packing material) (P1–8), respectively. Electrophoresis was carried out at a constant voltage of 80 V at 60°C for 12 h. Gels were stained with GoldViewer dye. A1–5: the Inlet, Sampling port 1–3, and the Outlet, correspondingly; P1–8: Packing 1–8, correspondingly.

**Figure 4:**
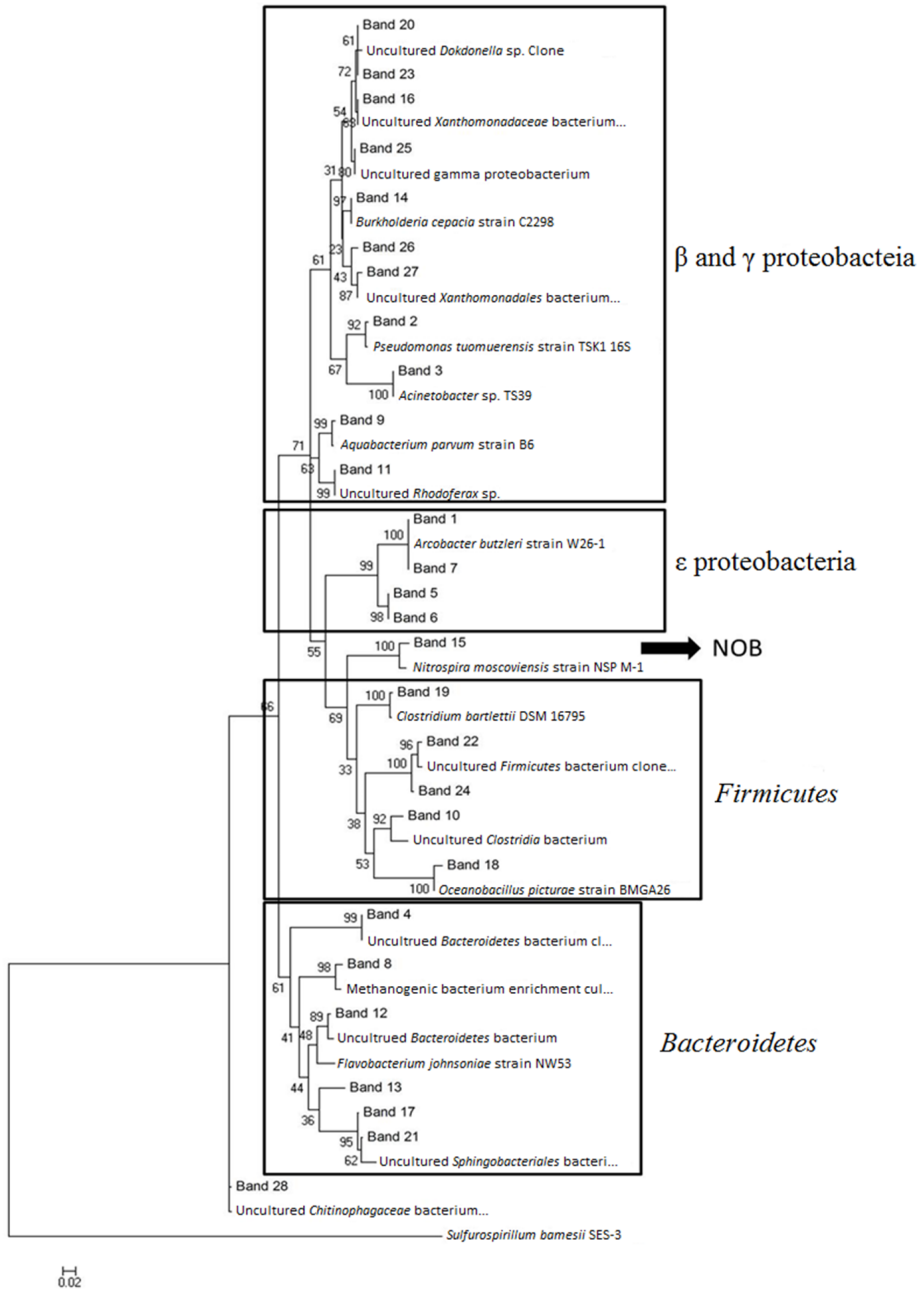
Phylogenetic analysis of 16S rRNA genes. Phylogenetic tree was constructed using the Neighbor-Joining method by MEGA v4.0.2 software. The numbers at the nodes are bootstrap values (*n* = 1000) and the Random seed value is 64,238.

Major DGGE bands of bacterial 16S rRNA gene were re-amplified and evaluated phylogenetically in order to identify putative functional bacteria in the flowing (water effluent) and the stationary phase (packing material) of the MRIS. DGGE profile of bacterial 16S rRNA gene showed frequent shifts in the composition and diversity of microbial community, as indicated by the appearance and disappearance of certain bands in different samples obtained from different sampling spots (Figure 3A). 28 bands were recovered and used for phylogenetic analysis (Figure 4). The relative abundance of the 16S rRNA genes indicated the richness of AOB in the MRIS. The Shannon index revealed that the community diversity between the flowing and the stationary phase did not vary significantly.

Phylogenetic analysis based on the retrieved 16S rRNA gene sequences showed generally four clusters (Figure 4). Three major groups affiliated to the phyla of proteobacteria (53.6%), firmicutes (17.9%), and bacteroidetes (21.4%), correspondingly. As in many WWTS, proteobacteria dominated the microbial population in the MRIS, among which β-, γ-, and ε-proteobacteria accounted for 10.7, 28.6, and 14.3% of the total clones, respectively. *Clostridia* sp. was simultaneously detected both in the flowing (band 10) and stationary phase (band 19). In addition, one band (15) was found to be directly relative to nitrification, which shared up to 97% sequence similarity to *Nitrospira moscoviensis*. Five bands (10, 18, 19, 22, and 24) exhibited the closest phylogenetic affinity to the firmicutes phylum. ε-proteobacteria were also detected and were clustered together: band 1 and 7 shared 100% sequence similarity to *Arcobacter butzleri*; band 5 and 6 shared 100% sequence similarity to *Sulfurospirillum barnesii*. The community diversity of AOB in the MRIS seems to be relatively low based on the DGGE profile of 16S rRNA gene.

### Analysis of community structure and composition of AOM based on *amo*A gene

Compared with 16S rRNA genes, detection of the *amo*A genes encoding the subunit of AMO could be more sensitive and specific for the analysis of community diversity and functional AOM abundance in the MRIS. Therefore, DGGE analysis based on specific ammonia oxidizing indicator *amo*A gene was conducted to assess the AOM community structure and composition. DGGE profiles based on AOB *amo*A gene exhibited nearly identical feature in all samples from the flowing phase, as shown by Figure 3B. The Shannon index showed that the AOB community of the stationary phase was more diverse than that of the flowing phase. Nine bands were selected for further BLAST and phylogenetic analysis (Figure 4), which revealed that 1) most of the closest relative sequences came from uncultured AOB; 2) the wastewater treatment plants rarely harbored *Nitrosospira* [24], nevertheless, bands obtained here exhibited great sequence similarity to *Nitrosospira* sp., band 1, 2 and 5 possessed up to 95, 99 and 96% sequence similarity to uncultured *Nitrosospira* sp. PJA1 (GenBank: DQ228457.1), *N. multiformis* ATCC 25196 (GenBank: DQ228454.1) and *Nitrosospira* sp. analogues (GenBank: GU136449.1), correspondingly, and band 3 showed 99% sequence similarity to *Nitrosolobus multiformis* analogue (GenBank: U91603.1); 3) all retrieved bands affiliated to β-proteobacterium (Table S1).

Three bands from DGGE profile of AOA *amo*A gene were successfully excised and sequenced (Figure 3C). BLAST analysis revealed that all the closest relative sequences deposited in GenBank came from moderately thermophilic AOA *Crenarchaeote*. Specifically, band 2 shared 99% sequence similarity to AOA discovered in acidic soil. In comparison with the relative stability of AOB community of the flowing phase, the AOA community of the flowing phase showed an increasing Shannon index along the depth of the filter sheet. However, the AOA diversity was relatively lower than the AOB diversity in the stationary phase (Figure 3B and Table S2).

### AOB and AOA abundance in the MRIS

**Figure 5:**
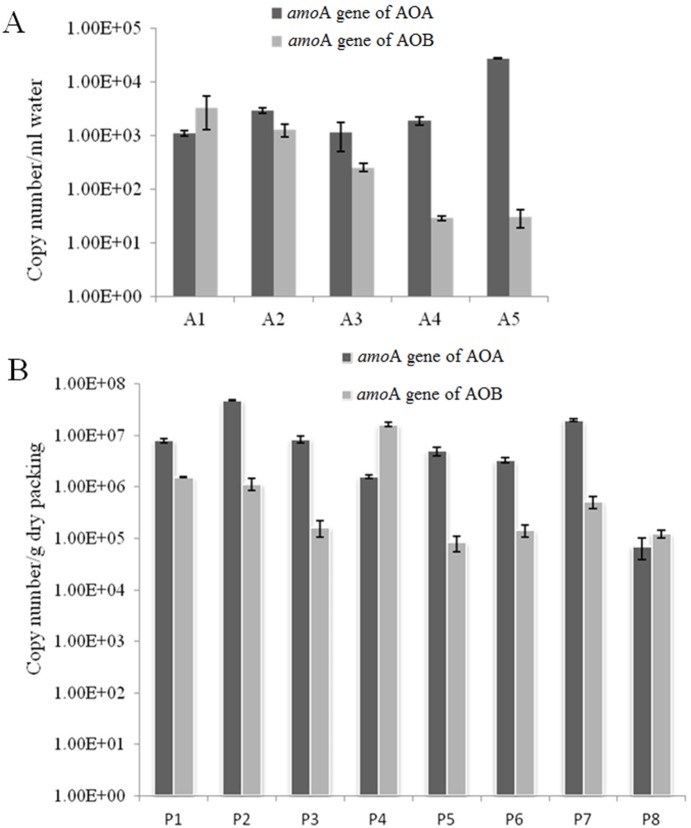
The copy numbers of AOB and AOA *amo*A genes from the flowing (A) and the stationary phase (B). All data are the means of values obtained from three parallel experiments ± SD (*t*-test, *p*<0.01) using the ΔΔCT method. A1–5: the Inlet, Sampling port 1–3, and the Outlet, correspondingly; P1–8: Packing 1–8, correspondingly.

Genetic diversity of AOB and AOA *amo*A genes from different samples was analyzed by qPCR. In the flowing phase, AOA abundance generally showed an imperfect increasing trend: it was relatively low in the Inlet (A1), but reached its peak in the Outlet (A5) (Figure 5A). The copy numbers of AOA *amo*A gene ranged from 1.11×10^3^ copies/mL to 2.75×10^4^ copies/mL (Figure 5A). However, AOB abundance showed an unambiguous decreasing trend along the depth of the filter sheet (Figure 5A). The copy numbers of AOB *amo*A *gene* ranged from 3.33×10^3^ copies/mL in the Inlet (A1) to 3.04×10^1^ copies/mL in the Outlet (A5). Furthermore, the abundance of AOA was greater than that of AOB in the flowing phase. However, the copy numbers of AOM *amo*A genes in the stationary phase fluctuated, no clear trend was observed. The copy numbers of AOA *amo*A genes ranged from 7.05×10^4^ to 4.82×10^7^ copies per gram dry weight, and that of AOB *amo*A genes from 8.31×10^4^ to 1.63×10^7^ copies per gram dry weight (Figure 5B). Neither AOA nor AOB abundance was significantly decrease or increase along with the depth of the filter sheet, and thus inconspicuous trend of AOB and AOA abundance was observed in the stationary phase (Figure 5B). Though the pH and ammonia decreased constantly (Figure 2), inconsistent trend of AOB copies displayed in the packing matrix was observed (Figure 5B). Such observation might be attributed to the different sizes of sands in the MIRS; moreover, the living bacteria proportion in the biofilm of the packing samples is hard to estimate.

### Effects of environmental factors on AOM communities

**Figure 6:**
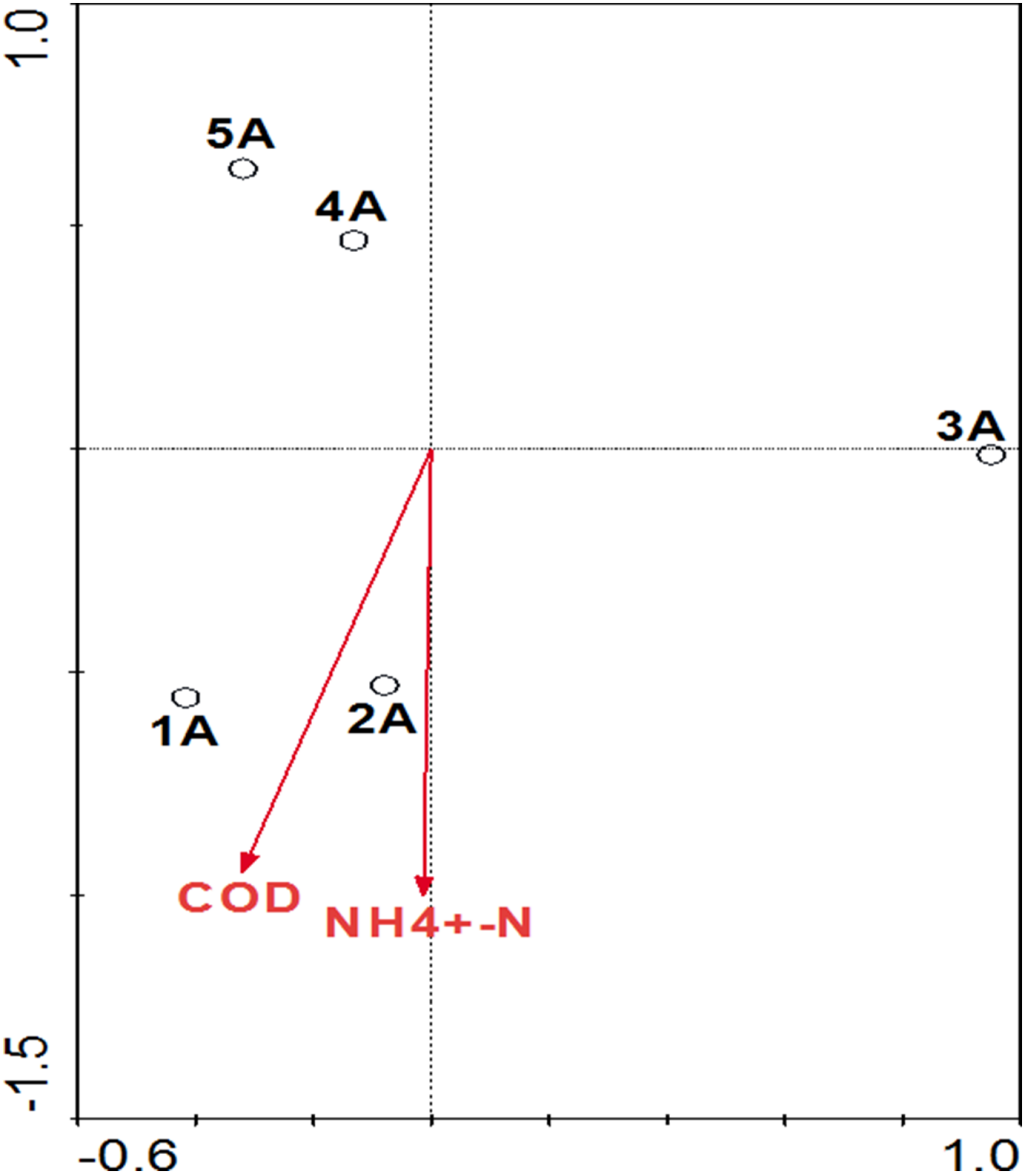
Triplot of RDA for AOB in the flowing phase. The first and the second axes explained 76.8% and 6.8% of the total variation respectively. The length of each arrow is correlated with the degree of relationship between the response variables. The arrows point in the direction of the maximum change for the associated variable. Open symbols represent samples from the flowing phase.

The relationship of the Shannon diversity index, *amo*A gene abundance and environmental variables was evaluated by SPSS 16.0. In the flowing phase, there was a significant positive correlation between DO concentration and AOA Shannon index (*r* = 0.963, *p*<0.05). Meanwhile, NH_3_-N showed a highly significant negative correlation (*r* = −0.998, *p*<0.01), whereas NO_3_-N showed a significant positive correlation (*r* = 0.965, *p*<0.05) with AOA Shannon index. No significant correlation was observed between AOB Shannon index and chemical indices in this study. Despite the fact that AOB abundance significantly correlated with DO concentration negatively (*r* = −0.998, *p*<0.01), significant positive correlation between AOB abundance and COD concentration (*r* = 0.997, *p*<0.01) was observed. However, there was no significant correlation between AOA community structure and environmental variables. When both COD and NH_3_-N were treated as environmental variables, the cumulative percent variance of the species-environment relationship indicated that the first and second canonical axes accounted for 76.8% and 6.8%, respectively. Moreover, Monte Carlo tests for the first axis and all canonical axes were significant (*p*<0.05) (Figure 6).

## Discussion

Effective ammonia removal in wastewater is mainly due to the combined effect of AOB and AOA. Certain environmental factors can affect both AOB and AOA abundance, such as ammonia, organic carbon, temperature, salinity, DO level, pH, nitrogen and carbon sources, sulfide, and phosphate, and so on [10], [24]. Among these factors, the ammonia level, salt concentration, DO density and pH value are the most important factors.

Microbial nitrification is a proton releasing reaction, which oxidizes ammonia to nitrite (NH_4_^+^ +2O_2_ → 2H^+^ + NO_3_^−^ + H_2_O). Accordingly, pH value along the depth of the filter sheet decreased as nitrification proceeded (Figure 2B). It was shown that nitrification could be down-regulated or even completely inhibited when the microenvironment pH was under 6.45 or above 8.95 [25]; nitrification in biofilms grown on chalk particles reached its peak at a pH around 4 owning to nitrifying populations related to subgroups with low *K_m_s* for ammonia [26]. However, it seemed that the oxidization of ammonia and nitrite in the MRIS maintained under extremely acidic condition (Figure 2). Evidence revealed that AOA were more predominant than AOB in acidic soils, where inhibition of AOB growth under acidic condition was observed [27]. AOB were sensitive to pH and could only thrive at neutral to alkali environments [24], but AOA were more tolerant to low pH. It appeared that AOA had a wide ecological and phylogenetic diversity under a broad range of pH [10]. For these reasons, a gradual decrease of AOB *amo*A gene abundance along the depth of the filter sheet could be expected in the MRIS, with an overall increase of AOA *amo*A gene abundance observed in the flowing phase (Figure 5A), where reducing ammonia and pH were monitored (Figure 2A and B). These results implied that AOA contributed more in the MRIS than AOB did.

It’s known that DO could suppress AOB activity at low concentration, resulting in limited nitrification [25] and leading to high COD [28]. Therefore, maximum AOB abundance (Figure 5A) in the superficial layers of the MRIS, where very low DO and high COD concentration were observed, might be attributed to the low DO concentration in this system (Figure 2A and B). Though considerable biomass, maximum AOB abundance and comparable AOA, was detected, NO_3_-N was undetectable because nitrification did not start up or was weak at the early stage in the superficial layers (Figure 2A). High DO led to oxidization of nitrite to nitrate and inefficient denitrification [29], causing nitrate accumulation in the Outlet (A5). Therefore, suppression of AOB with increasing nitrate was observed, when DO increase along the depth of the filter sheet (Figure 2A).

AOA community diversity in the MRIS (Figure 3C) positively correlated with DO concentration as indicated by Shannon index (*r* = 0.963, *p*<0.05), Meanwhile, NH_3_-N displayed a significantly negative correlation (*r* = −0.998, *p*<0.01) with DO concentration. These results implied that DO and NH_3_-N were two important factors for shaping AOA community. The changes of AOA community structure in the flowing phase also demonstrated that the influent wastewater fed the packing (stationary phase) to form biofilms and led to partial cell accumulation. Though no significant correlation was observed between AOB Shannon index and chemical indices, a close association of RDA indicated that COD and NH_3_-N were best correlated with the AOB distribution in the MRIS (Figure 6), as RDA showed that the selected parameters were fine explanatory variables of the AOB community structure.

Ammonia has two opposite effects on the growth of AOB. High ammonia promotes AOB growth, while low ammonia inhabits its growth [30]. In contrast to AOB, AOA can endure very low ammonia concentration [31]. Along the depth of the filter sheet, the growth of AOB was suppressed but that of AOA was strengthened (Figure 5A) when ammonia concentration decreased (Figure 2A). In soil samples, AOB and AOA also preferred different N conditions for growth: AOB required high ammonia, while AOA required low ammonia [32]. Similar case in this study revealed different contribution of AOB and AOA to N cycling in the MRIS, AOA may play more important role in ammonia oxidization in the flowing phase. When DO level was taken into consideration, confusion arose to some extent due to the reason that AOA were generally thought to be more active in low-oxygen and oxic-anoxic surroundings [33], while most AOB preferred aerobic condition [34]. Thought significant negative correlation between AOB abundance and DO concentration (*r* = −0.998, *p*<0.01) and significant positive correlation between AOB abundance and COD concentration (*r* = 0.997, *p*<0.01) were observed, DO level seemed to be a relative “trivial” factor for AOA in the MRIS, as both DO and COD concentration had no significant correlation with AOA abundance. Yet, fully aerobic growth of *Nitrosopumilis maritimus* during cultivation and near-stoichiometric conversion of ammonium to nitrite were reported [35]. Thus we could not rule out the possibility that some aerobic AOA might exist in the MRIS and contribute to nitrification in spite of ‘toxic’ oxygen. The population feature in the stationary phase was characterized by divergent expression level of *amo*A genes (Figure 5B). In combination with AOM abundance in the flowing phase, the copy numbers of AOA *amo*A genes exceeded those of AOB in the MRIS, as microbial nitrification went on along the depth of the filter sheet.

Phylogenetic analysis based on 16S rRNA gene sequences showed relative similarity to the rotating activated bacillus contactor (RABC) [36], where three major groups were found (Figure 4). Among the firmicutes phylum, *Clostridia* sp. was found both in the flowing (band 10) and the stationary phase (band 19) implying its important role in the removal of organic waste in the MRIS. Two bands (band 5 and 6) belonging to *S. barnesii* SES-3 were also found. *S. barnesii* is frequently found in wastewater to simultaneously remove selenite and nitrate [37], [38]. Only one band (15) directly relative to nitrification was found (Figure 4), which shared 97% sequence similarity to *N. moscoviensis* NSP M-1. However, no band sharing high similarity to ammonia oxidizers was found. This might be attributed to the population of AOB is too less to be detected, or the primers used for universal bacterial 16S rRNA genes did not match well with AOB template.

Due to no band retrieved from DGGE profile of bacterial 16S rRNA gene showed sequence similarity to ammonia oxidizers, making it difficult to elucidate the abundance and diversity of ammonia oxidizers. Therefore detection of the *amo*A gene encoding the subunit of AMO was necessary. BLAST analysis of AOB *amo*A genes found that band 1, band 2, band 5 shared 95, 99, and 96% sequence similarity to *Nitrosospira*, respectively (Figure 3B and Table S1). *Nitrosospira* sp. was vital for ammonia-oxidizing activity in acid soils, the community shift of *Nitrosospira* population was found to be pH-associated [32], [33]. Though the pH value of the stationary phase (P1–8) had not yet been detected in this article, the niche of P5–8 might be acidic because pH in the flowing phase dramatically decreased along the depth of the filter sheet (Figure 2B). Actually, a pH-associated trend might exist as implied by the distinctive appearance of these bands: band 1 was found only in P5 and P6; band 2 was found in all samples, especially in P4 and P5; band 5 was found in P6 and P7.

From the AOM abundance we could draw the conclusion that both AOB and AOA contributed to nitrification in the MRIS. The presence of distinctive phylotypes and the relatively higher ratio of archaeal vs. bacterial transcriptional activity implied that autotrophic ammonia oxidation in this system might be attributed mainly to archaea. The indistinctive dominance of AOA over AOB might correlate with different microenvironments in different layers of the MRIS. Further research should be implemented to link various environment factors that shape the diversity and abundance of AOB and AOA.

## Supporting Information

Table S1 **AOB** ***amo*** **A genes retrieved from DGGE profile.**
(DOC)

Table S2 **AOA** ***amo*** **A genes retrieved from DGGE profile.**
(DOC)
