# Correction: Ammonia Oxidizers in a Pilot-Scale Multilayer Rapid Infiltration System for Domestic Wastewater Treatment

**DOI:** 10.1371/journal.pone.0118656

**Published:** 2015-02-25

**Authors:** The PLOS ONE Staff

There are errors in the author affiliations. The affiliations should appear as shown here:

Yingli Lian^1,2^, Meiying Xu^2, 4*^, Yuming Zhong^2,4^, Yongqiang Yang^3^, Fanrong Chen^3^, Jun Guo^2,4^

1. School of Biological Science & Engineering, South China University of Technology, Guangzhou, 510006, China.

2. Guangdong Provincial Key Laboratory of Microbial Culture Collection, Guangdong Institute of Microbiology, Guangzhou, 510070, China.

3. Guangzhou Institute of Geochemistry, Chinese Academy of Sciences, Guangzhou, 510640, China.

4. State Key Laboratory of Applied Microbiology Southern China, Guangzhou, 510070, China.
