# Supplementary material for: Ammonia Oxidizers in a Pilot-Scale Multilayer Rapid Infiltration System for Domestic Wastewater Treatment
Source: PLoS One. 2014 Dec 5;9(12):e114723. doi: 10.1371/journal.pone.0114723 (PMC4257731; doi:10.1371/journal.pone.0114723)
Supplement: Table S1 — AOB amo A genes retrieved from DGGE profile. (DOC) [file pone.0114723.s001.doc]

**Table S1.** AOB *amoA* genes retrieved from DGGE profile

| Retrieved band | Closest relative NO.  (accession number) | Identity (%) | Genera |
| --- | --- | --- | --- |
| Band1 | DQ228457.1 | 95% | *Nitrosospira sp.* PJA1 |
| Band2 | DQ228454 | 99% | *Nitrosospira multiformi*s ATCC 25196 |
| Band3 | U91603.1 | 94% | *Nitrosolobus multiformis* |
| Band4 | AY353583.1 | 99% | Uncultured ammonia-oxidizing bacterium |
| Band5 | GU136449.1 | 96% | Uncultured *Nitrosospira sp.* |
| Band6 | AY353581.1 | 98% | Uncultured ammonia-oxidizing bacterium |
| Band7 | HQ215929.1 | 99% | Uncultured ammonia-oxidizing bacterium |
| Band8 | AB635455.1 | 100% | Uncultured ammonia-oxidizing bacterium |
| Band9 | HM461394.1 | 99% | Uncultured ammonia-oxidizing bacterium |
