# Supplementary material for: Ammonia Oxidizers in a Pilot-Scale Multilayer Rapid Infiltration System for Domestic Wastewater Treatment
Source: PLoS One. 2014 Dec 5;9(12):e114723. doi: 10.1371/journal.pone.0114723 (PMC4257731; doi:10.1371/journal.pone.0114723)
Supplement: Table S2 — AOA amo A genes retrieved from DGGE profile. (DOC) [file pone.0114723.s002.doc]

**Table S2.** AOA *amoA* genes retrieved from DGGE profile

| Retrieved band | Closest relative NO.  (accession number) | Identity (%) | Genera |
| --- | --- | --- | --- |
| Band 1 | JQ320519.1 | 99% | Uncultured crenarchaeote |
| Band 2 | JF681783.1 | 99% | Uncultured archaeon |
| Band 3 | JN179669.1 | 96% | Uncultured archaeon |
